# Very low intensity ultrasounds as a new strategy to improve selective delivery of nanoparticles-complexes in cancer cells

**DOI:** 10.1186/s13046-018-1018-6

**Published:** 2019-01-03

**Authors:** Rossella Loria, Claudia Giliberti, Angelico Bedini, Raffaele Palomba, Giulio Caracciolo, Pierpaolo Ceci, Elisabetta Falvo, Raffaella Marconi, Rita Falcioni, Gianluca Bossi, Lidia Strigari

**Affiliations:** 1 Department of Research, Advanced Diagnostics and Technological Innovation, Area of Translational Research, IRCCS - Regina Elena National Cancer Institute, Rome, Italy; 2 Dipartimento Innovazioni Tecnologiche e Sicurezza degli Impianti, Prodotti e Insediamenti Antropici (DIT), INAIL, Rome, Italy; 3 Department of Molecular Medicine, “Sapienza” University of Rome, Rome, Italy; 4 Institute of Molecular Biology and Pathology, CNR, Rome, Italy; 5 Laboratory of Medical Physics and Expert Systems, IRCCS - Regina Elena National Cancer Institute, Rome, Italy

**Keywords:** Ultrasound, Nanoparticles, Chemotherapeutic drugs, Sarcoma, Colon cancer

## Abstract

**Background:**

The possibility to combine Low Intensity UltraSound (LIUS) and Nanoparticles (NP) could represent a promising strategy for drugs delivery in tumors difficult to treat overcoming resistance to therapies. On one side the NP can carry drugs that specifically target the tumors on the other the LIUS can facilitate and direct the delivery to the tumor cells. In this study, we investigated whether Very Low Intensity UltraSound (VLIUS), at intensities lower than 120 mW/cm^2^, might constitute a novel strategy to improve delivery to tumor cells. Thus, in order to verify the efficacy of this novel modality in terms of increase selective uptake in tumoral cells and translate speedily in clinical practice, we investigated VLIUS in three different in vitro experimental tumor models and normal cells adopting three different therapeutic strategies.

**Methods:**

VLIUS at different intensities and exposure time were applied to tumor and normal cells to evaluate the efficiency in uptake of labeled human ferritin (HFt)-based NP, the delivery of NP complexed Firefly luciferase reported gene (lipoplex-LUC), and the tumor-killing of chemotherapeutic agent.

**Results:**

Specifically, we found that specific VLIUS intensity (120 mW/cm^2^) increases tumor cell uptake of HFt-based NPs at specific concentration (0.5 mg/ml). Similarly, VLIUS treatments increase significantly tumor cells delivery of lipoplex-LUC cargos. Furthermore, of interest, VLIUS increases tumor killing of chemotherapy drug trabectedin in a time dependent fashion. Noteworthy, VLIUS treatments are well tolerated in normal cells with not significant effects on cell survival, NPs delivery and drug-induced toxicity, suggesting a tumor specific fashion.

**Conclusions:**

Our data shed novel lights on the potential application of VLIUS for the design and development of novel therapeutic strategies aiming to efficiently deliver NP loaded cargos or anticancer drugs into more aggressive and unresponsive tumors niche.

**Electronic supplementary material:**

The online version of this article (10.1186/s13046-018-1018-6) contains supplementary material, which is available to authorized users.

## Background

Cancer is a leading cause of death worldwide [1]. Tumor heterogeneity is the main cause of resistance to therapeutic treatments due to the selection of surviving cancer cells that, becoming resistant to therapies and dominant in the tumor, are potentially responsible for recurrence [2]. Surgical resection is the mainstay of treatment for localized disease, while combined treatments may change the natural history of more aggressive tumors. Unfortunately, few therapeutic options are available for aggressive local or metastatic diseases (sarcoma/liposarcoma or colon cancer) which are generally associated with a poor prognosis. Benefits of adjuvant and neoadjuvant chemotherapy in advanced disease are still debated due to potential toxic side effects on normal tissues [3] and diverse sensitivity and response to chemotherapy with the tumor subtypes [4] potentially leading to death of many patients. Accordingly, the identification of adequate and innovative treatments to moderate toxic side effects occurrence, improve therapy efficiency and ameliorate quality of life and life expectancy in cancer patients is demanding.

In this context, focused ultrasound (US) represents a non-invasive technology that can be adopted for local tumor ablation deep inside the body without causing severe harm to overlying skin and adjacent normal tissues. Of interest, during the last years, low to medium intensity US was revealed as compelling tool for the improvement of several emerging therapeutic applications [5–8]. Indeed, the capability of pulsed US in transferring mechanical energy through the different layers of the skin and underlying tissues, generating temporary non-lethal porosity in cell membrane, known as sonoporation [9], enhances cellular membrane permeability constituting an intriguing and novel therapeutic option for more efficient strategies for gene and/or drug delivery [10].

Nanoparticles (NPs) constitute a novel not hazardous non-viral vehicle, for the delivery, by encapsulation, of nucleic acid (DNA, siRNA) and/or therapeutic compounds that may otherwise cause systemic toxicity if delivered in free form. Various types of NPs have been intensively investigated for increasing local tumor delivery [11–14]. In particular, protein-cage molecules based on ferritins (Fts) are attracting growing interest in the field of drug-delivery, due to their exceptional characteristics, namely biodegradability, solubility, functionalization versatility and remarkable capacity to bind different types of drugs [15]. Nevertheless, albeit the outstanding potentiality, the identification of strategies aimed to improve uptake and delivery of therapeutic NPs in the tumor site are still highly desired.

Of interest, low-intensity US (LIUS) have been shown to enhance the delivering of liposomal drug carriers in cancer cell increasing their therapeutic efficacy [16]. To date a widely accepted definition of LIUS is missing, and most of the studies in cancer cells have been generally performed with intensity lower than 5.0 W/cm^2^, corresponding to a root-mean-square pressure amplitude of about 0.3 MPa [17]. Very low intensity of non-cavitational US (VLIUS) has been reported to allow the internalization of small drugs model molecules when higher time of exposure are used in NIH murine fibroblast-like culture (NIH-3 T3) [18].

In this study, we investigated whether VLIUS at intensities, to induce sonoporation at subcavitational levels, lower (0.04, 0.08 0.12 W/cm2) than that already reported [19–21] could constitute a novel approach to improve delivery of therapeutic compounds in tumors of different type (sarcoma and colon). At the best of our knowledge, no internalization studies have been performed using a low intensity megasonic field. Accordingly, in order to verify the efficacy of this novel modality in terms of increase selective uptake in tumoral cells and translate speedily in clinical practice, we investigated VLIUS in three different in vitro experimental tumor models and normal cells adopting three different therapeutic strategies. We demonstrated that VLIUS enhances delivery of NPs and chemotherapy drug in cancer cells at the experimental conditions adopted without significant effects in normal cells.

## Methods

### Cell lines

The human lines colon adenocarcinoma (HT29), colorectal carcinoma (HCT116), human fibroblast (HF), endothelial umbilical vein (E926), and sarcoma (SW872 and SW982, provided by ATCC) were all cultured in DMEM (Dulbecco’s modified Eagle’s medium, Eurobio, Les Ulis, France), supplemented with 10% heat-inactivated FBS (Gibco, Life technologies, Milan, Italy), 1% penicillin/streptomycin and 1% Glutammine (Gibco, Life technologies, Milan, Italy). The myxoid sarcoma lines 402–91 WT [22] and the resistant counterpart 402–91 ET [23] were maintained in RPMI medium supplemented with 10% heat-inactivated FBS and 1% penicillin/streptomycin (Gibco, Life technologies, Milan, Italy). All lines grow at 37 °C in a humidified atmosphere with 5% CO_2_.

### Production of HFt-based NPs

Recombinant H-type human ferritin (HFt) and fluorescein-labelled (HFt-FITC) were prepared as described previously [24].

### Production of lipoplex-LUC

Zwitterionic helper lipids dioleoylphosphatidylethanolamine (DOPE) and dioleoylphosphocholine (DOPC) and the monovalent cationic lipids 1,2-dioleoyl-3-trimethylammonium-propane (DOTAP) and (3β-[N-(N′,N′-dimethylaminoethane)-carbamoyl])-cholesterol (DC-Chol) were purchased from Avanti Polar Lipids (Alabaster, AL, USA) and used without further purification. According to standard protocols, lipid vesicles (DOPE, DOPC, DOTAP and DC-Chol) were dissolved in chloroform at the desired molar ratio (3:1:1:3) (patent number RM2012A000480). The solvent was evaporated under vacuum for at least 24 h and obtained lipid films hydrated with Tris-HCl (10 mM, pH 7.4) to achieve the desired final lipid concentration (1 mg/mL). Lipid dispersions were sonicated to clarity to prepare small unilamellar vesicles (SUVs). Protamine sulfate salt (P) from salmon (MW = 5.1 kDa) was purchased from Sigma-Aldrich (St. Louis, MO, USA) and dissolved in ultrapure water (final concentration = 1 mg/ml). For each well, negatively charged P/DNA microspheres were prepared by mixing 1.25 μg of P with 2.5 μg of DNA vector pGL4.51-LUC-CMV-Neo (weight ratio, R_W_ = 0.5, zeta potential = − 19.5 ± 2.5 mV). After 20 min incubation, lipid/DNA NPs were prepared by mixing negatively charged P/DNA microspheres with lipid vesicles at cationic lipid/DNA charge ratio, ρ = 3. After pipetting up and down a few times, lipoplex-LUC were kept at room temperature for 15–30 min before use.

### Size and zeta-potential measurements

The lipoplex-LUC were highly homogeneous (polydispersity index = 0.11 ± 0.02), small in size (R_H_ = 255 ± 23 nm) and positively charged (ζ_P_ = 32.3 ± 11.2 mV). Hydrodynamic radius (R_H_) and zeta-potential (ζ_P_) distributions of lipid/DNA NPs were measured at 25 °C by a ZetaSizer spectrometer (Malvern, UK) equipped with a 5 mW He−Ne laser (wavelength λ = 632.8 nm) and a digital logarithmic correlator. The normalized intensity autocorrelation functions were analyzed by a dedicated software, which allows obtaining the distribution of the diffusion coefficient D of the particles. This coefficient is converted into an effective hydrodynamic radius R_H_ by using the Stokes–Einstein equation R_H_ = K_B_T/ (6πηD), where K_B_T is the thermal energy and η the solvent viscosity. R_H_ and ζ_P_ are reported as the average ± standard deviation (s.d.) of three independent measurements.

### VLIUS setup

**Fig. 1:**
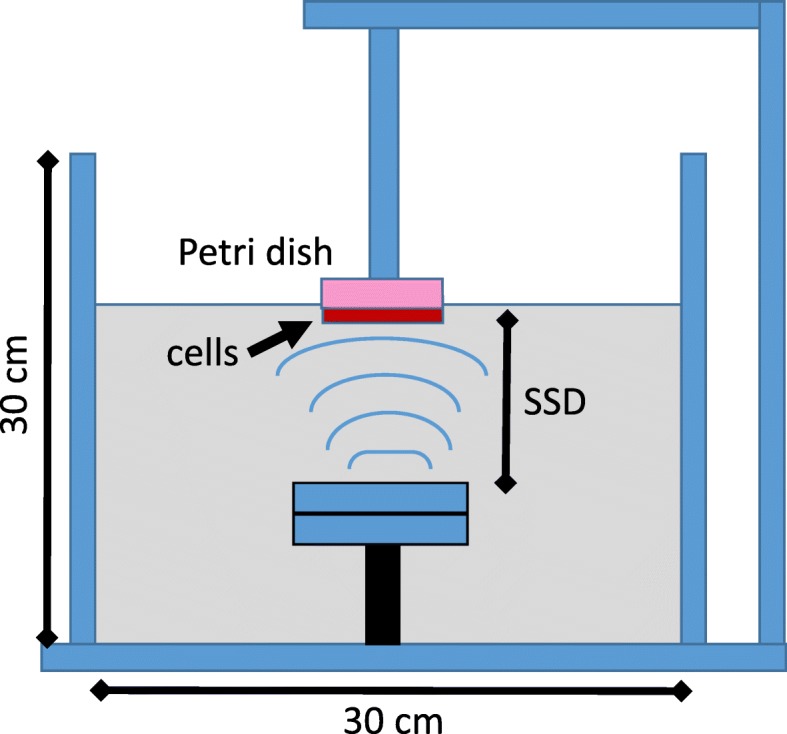
Ultrasounds setup. For VLIUS exposure, a line of ultrasonic signal was generated by a piezoelectric unfocussed transducer immersed at the bottom of a tank filled with degassed water, powered to a signal generator (Agilent 33220A) and a signal amplifier (Amplifier Research 25A250). A sinusoidal signal at the frequency of 1 MHz was generated and measured by a needle hydrophone (S.N. 2090, Precision Acoustics) of 0.5 mm diameter with a sensitivity of 483 mV/MPa at 1 MHz, connected to an oscilloscope (Tektronix TDS 3052B). Continuous ultrasound exposures in terms of ‘spatial peak temporal average intensity’ (Ispta) equal to 40, 80, 120 and 150 mW/cm^2^ were administered for 15 min on a petri dish (60 mm), submerged up to half of its thickness and aligned coaxially with the transducer, at a fixed distance (Source-dish Surface Distance (SSD) from the transducer

Treatments were performed with a homemade device (Fig. 1) [18] consisting of a signal generator (Agilent 33220A), a signal amplifier device (Amplifier Research 25A250) combined with a sine wave oscillator together with waterproof ultrasonic piezoresistive unfocussed transducer (S.N. PA517, Precision Acoustics, UK, 6 cm diameter) immersed at the bottom of a tank (30 × 30 × 30 cm) filled with degassed Milli-Q water (18.2 MΩ·cm, resistivity). Ultrasonic transducer is engineered to be stimulated in burst or continuous regimes under drive signal amplitude of 10–100 V, in the 1–5 MHz frequency range, providing the main therapeutic ultrasound settings. A sinusoidal signal at the frequency of 1 MHz was generated and measured by a needle hydrophone (S.N. 2090, Precision Acoustics) of 0.5 mm diameter with a sensitivity of 483 mV/MPa at 1 MHz, connected to an oscilloscope (Tektronix TDS 3052B). Continuous ultrasound exposures in terms of spatial peak temporal average intensity (Ispta) equal to 40, 80, 120 and 150 mW/cm^2^ were administered for fixed time-points to hermetically sealed cell culture 60 mm Petri dish containing cells with 3.0 ml of growth media. Plate was positioned above the transducer at the water surface submerged up to half of its thickness aligned coaxially with the transducer, at a fixed distance Source-dish Surface Distance (SSD) of 12 cm from the transducer as reported in Fig. 1. The temperature of the water bath was monitored by thermocouple system (Lutron electronic enterprise co., LTD.) and kept constantly at 25 °C (accuracy, ±1 °C) both inside and outside the Petri dish.

### HFt-based NPs delivery

SW872 and SW982 cells were plated at density of 2,0 × 10^5^ cells on poly-l lysine coated slides in 60 mm dish. The day after cells were incubated for 1 h with HFt-based NPs plus or minus 15 min exposure to VLIUS at different intensities (40–120 mW/cm^2^). Following cells were counterstain with Hoechst (SIGMA-Aldrich) and analyzed under Microscope OLYMPUS BX53 for immunofluorescence dots. Each experiment was carried-out in quadruplicate and repeated at least three times.

### The lipoplex-LUC delivery

Either HT29, HCT116, HF, or E926 cells were plated in 60 mm dishes at density of 5,0 × 10^4^ cells/dish. The day after cells were replenished with OPTIMEM, and exposed to VLIUS at intensity 120 mW/cm^2^ for different time lengths (5, 10, 15, 20 min). The lipoplex-LUC cargos were delivered to the cells right-before or right-after VLIUS treatments. The day after culture media was replaced with regular grow media. Then, 48 h later cells were collected, rinsed with PBS and lysed with 200 μl of Passive Lysis Buffer (PLB; Cat.#E1941 Promega). Protein lysates were clarified by centrifugation (12,000 RPM × 15 min + 4 °C) and 30 μl of collected supernatants incubated in triplicate with Luciferase Assay Reagent (Promega) before reading to GloMax® 96 Microplate Luminometer. Values were normalized to protein concentration for each sample. Each experiment was carried-out in triplicate and repeated at least three times.

### Drug treatments

Trabectedin kindly provided by PhamaMar S.A (Colmenar Viejo, Spain) was stored at − 20 °C in DMSO at a concentration of 1 mM, and diluted in RPMI before treatment. HF, E926, 402–91 WT and 402–91 ET cells were plated at density of 1,5 × 10^5^ cells in 60 mm dish, and the day after treated for 1 h with trabectedin at a concentration of 10 or 25 nM. During the treatment cells were also exposed 1, 5, 10 or 15 min to VLIUS at different intensities of 20 or 40 or 80 mW/cm^2^. Cell vitality was evaluated by Crystal violet staining 48 h after drug removal. Each experiment was carried-out in triplicate and repeated at least three times.

### Image analysis

The database of available fluorescence images was divided in training (one of the images at 40 and 80 mW/cm^2^) and investigation (the remaining) dataset. A Matlab tool was developed to import each image and split it into three images, one for each RGB channel. Based on the histogram and profiles carried-out on the green channel of training dataset, a cut-off of 10 was set as threshold. A visual inspection of green images and of each histogram was performed based on the identified cut-off to verify that green areas correspond to investigated cells. The Matlab function “regionprops” was used to extract the area and the eccentricity of identified regions. The fraction of pixels over the cutoff of 10 was calculated as the ratio between the sum of counts over the cuf-off and the original green images and calculated for each image. The standard deviation of the measured fractions was determined in the images for each experimental condition. The fractions and the error bars were plotted according to each experimental setup.

### Statistical analysis

Data were reported as mean and standard deviation. All analyses were performed using one-way/two-way ANOVA and Dunnett’s /Tukey’s multiple comparisons post-hoc test as appropriate. Differences were considered statistically significant when *P* ≤ 0.05.

## Results

### VLIUS improves HFt-based NP cellular uptake in sarcoma cells in vitro

**Fig. 2:**
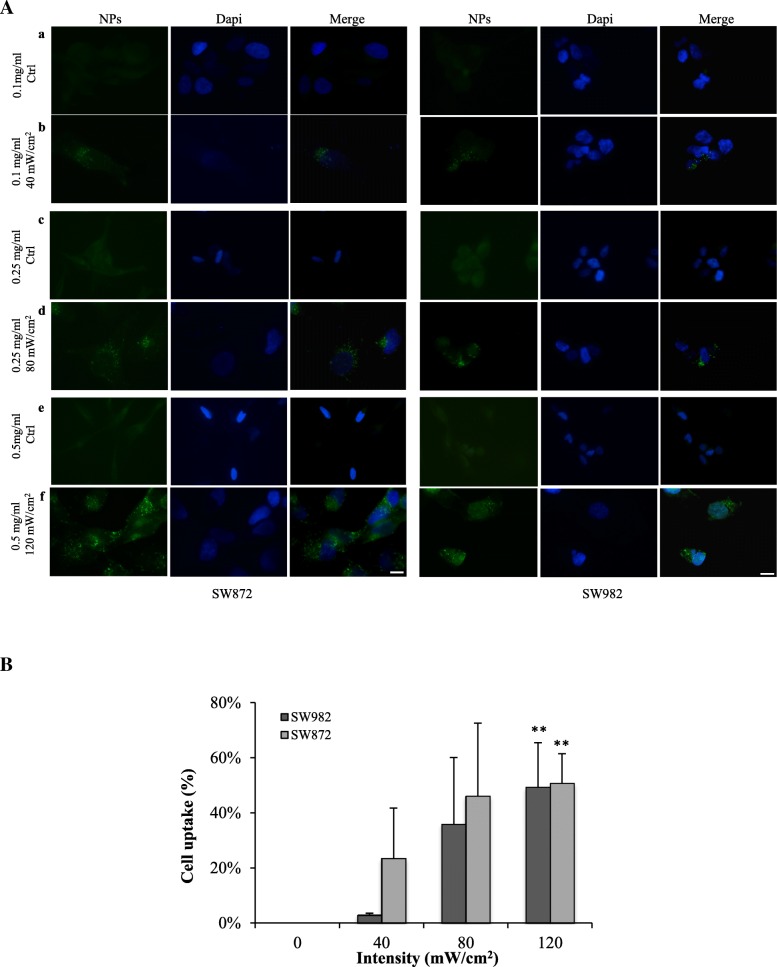
VLIUS treatments increases HFt-based NPs cell uptake. **a** SW872 and SW982 cells were plated on poly-l lysine coated slides and the day after incubated for 45 min in the presence of HFt-based NPs at different concentration (0.1–0.25 – 0.5 mg/ml) and following exposed 15 min to VLIUS source with increasing intensities (40–80 – 120 mW/cm^2^). Control cells were treated 1 h with HFt-based NPs alone. After incubations cells were counterstained with Hoechst to highlight nuclei and analyzed for immunofluorescence dots. Fluorescence dots were quantified as fraction of pixels over the cutoff of 10 using in the green channel of each image. Scale bar is 10 μm. **b** Histogram reported percentage of uptake and error bars represent the standard deviation of the measured fractions in the images for each experimental condition. Each experiment was carried out in quadruplicate and repeated at least three times. Significance was assessed by using two-way ANOVA and Tukey’s multiple comparisons post-hoc test. * *P* < 0.05, **P < 0.001, ***P < 0.0001

In order to investigate VLIUS effects on endocytosis and thus cellular uptake in sarcoma cells, we quantified the subcellular localization of fluorescent dots generated by HFt-based NPs delivery upon exposure to a VLIUS source generated with a homemade device (Fig. 1). To this aim, adherent cell cultures from two different sarcoma lines (SW872, SW982) were incubated with HFt-based NPs at three different concentrations (0.1–0.25 – 0.5 mg/ml) and thereafter exposed for 15 min to a VLIUS source with constant 1.0 MHz frequency and increasing intensities (40–80 - 120 mW/cm^2^). Cells which received HFt-based NPs without VLIUS were adopted as negative control. Right after incubations cells were stained and analyzed at single cell level under microscope to quantify fluorescence dots. Results revealed that VLIUS exposure enhance HFt-based NPs delivery in both tested lines (Fig. 2). The most efficient cellular uptake was achieved when the highest HFt-based NPs concentration (0.5 mg/ml) was combined to the maximum LIUS intensity (120 mW/cm^2^) (Fig. 2a, b). Of interest, no fluorescence dots were observed in control cells challenged with HFt-based NPs without VLIUS (Fig. 2a). Fluorescence dots were homogenously distributed in the cytosol with no nuclear staining, and cell viability was close to 100% in all experimental settings explored (data not shown) supporting the effectiveness of VLIUS in promoting NPs delivery in tumor site.

### VLIUS increases lipoplex-LUC delivery in colon cancer cells in vitro

**Fig. 3:**
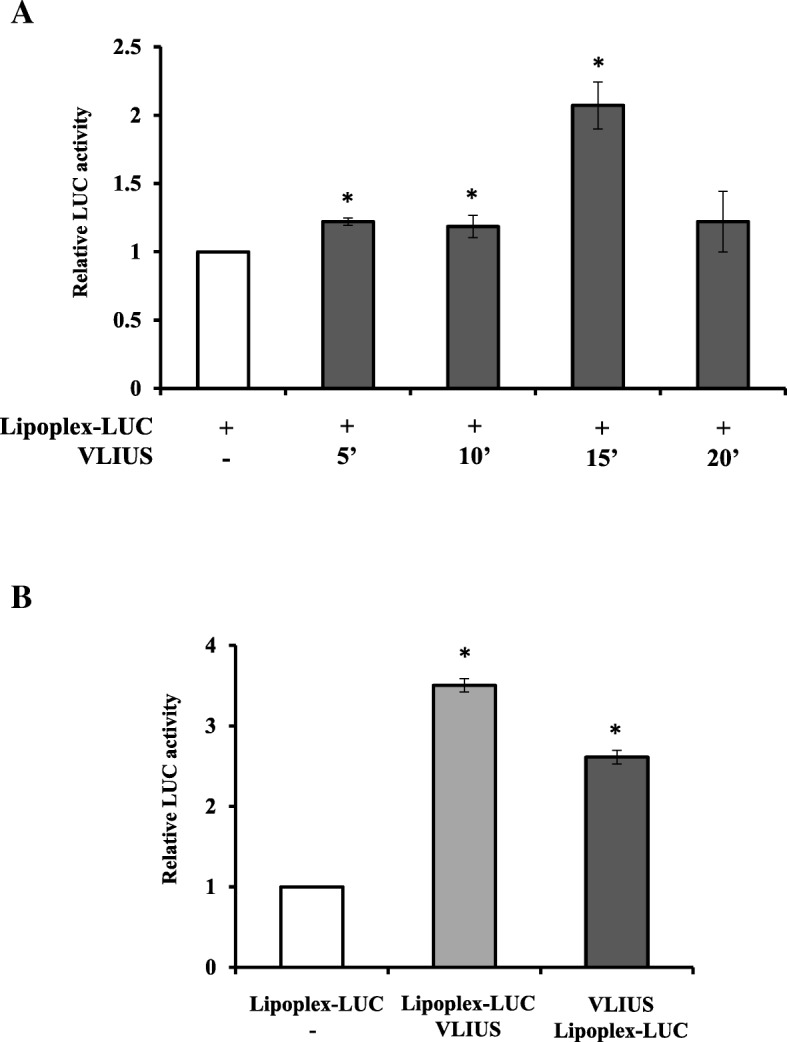
US exposure enhances cellular uptake of LipoplexLUC NP complexes. **a** HT29 cells plated at density of 5.0 × 10^5^ cells in 60 mm dish, were delivered with lipoplex-LUC cargos and right after exposed to VLIUS source for the indicated times lengths. **b** HT29 cells plated as reported in A. The day after cells where either delivered with lipoplex-LUC cargos and treated with VLIUS for 15 min (Lipoplex-LUC / VLIUS) or with opposite schedule VLIUS for 15 min and then incubated lipoplex-LUC cargos (VLIUS / Lipoplex-LUC). In all treatments reported in (**a** and **b**) cells were collected 48 h after treatments. DNA delivery efficiency was assessed by luciferase assays and values were normalized to protein content and relative LUC activity quantified with respect to control set to 1.0. Each experiment has been repeated three times in triplicate, means and standard deviation of representative experiments are reported. Significance was assessed by one-way ANOVA and Dunnett’s multiple comparisons post-hoc test. *P < 0.05, **P < 0.001, ***P < 0.0001

In order to assess whether VLIUS might increase transfer of DNA-loaded NPs in cancer cells [25], we investigated in hard-to-transfect HT29 colon cancer cells [26] the delivery efficiency of DNA vector encoding Firefly luciferase reporter (LUC) gene complexed with lipid vesicles (lipoplex-LUC). Luciferase-based technology allows to record the average of reporter expression in the whole cell population. To define the most suitable VLIUS wave, we took advantage from previously reported analyses (Fig. 2) and selected the US frequency and intensity that showed the highest HFt-based NPs internalization (1,0 MHz frequency and 120 mW/cm2 intensities). We first investigated VLIUS by treatments with different time-length exposure to optimize cell permeability and hence delivery. Thus, HT29 cells were incubated with equal amount of lipoplex-LUC cargos and left untreated or incubated with VLIUS at established intensity varying the exposure time (5, 10, 15, 20 min), and cell delivery efficiency evaluated 48 h later. VLIUS at 15 min exposure time was revealed as the most efficient to deliver lipoplex-LUC cargos in HT29 cells when compared to all other tested treatments (Fig. 3a). Results observed with longer VLUIS exposure time (20 min) might depend to the exit of lipoplex-LUC cargos from the cells likely due to a protracted exposure as reported [27].

We next investigated whether different schedule of treatment might improve DNA delivery in HT29 cells, and lipoplex-LUC cargos were added to the cells immediately before or immediately after the established VLIUS treatment (120 mV/cm^2^, 15 min). When compared to untreated cells, VLIUS treatment increases significantly lipoplex-LUC internalization in both tested conditions, however, a significantly higher DNA delivering was observed when lipoplex-LUC cargo were added to the cells before VLIUS treatments, in tested cancer cells (Fig. 3b). Hence, the therapeutic strategies modelling could constitute a crucial procedure to identify optimal setting for more efficient compounds delivery.

### VLIUS increases lipoplex-LUC delivery in cancer but not in normal cells

**Fig. 4:**
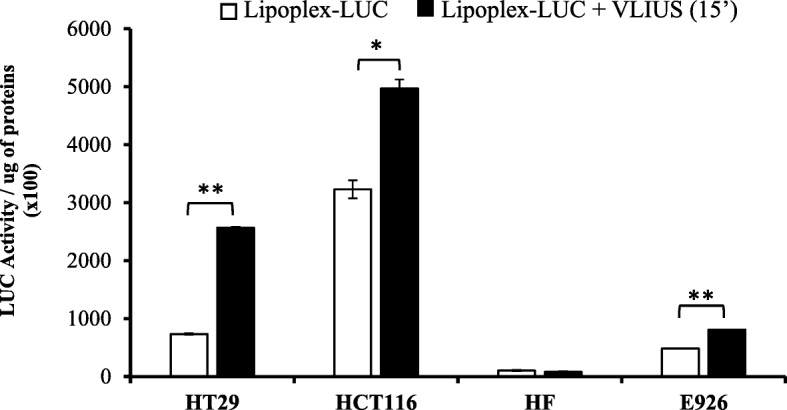
Ultrasound pre-treatment enhances significantly cellular NP-DNA complexes uptake in cancer cells but not normal cells. Cancer HT29, HCT116 and normal HF, E926 cells were plated (5.0x10^5^cells / dish) in 60 mm dishes, then twenty-four hours later, growth media was replaced with OPTIMEM, delivered with liploplex-LUC complexes and thereafter ultrasounds treated for the indicated times. Cells were collected 48 h after treatments. DNA transduction efficiency was evaluated by luciferase assays and values were normalized to protein content and relative LUC activity quantified respect to control set to 1.0. Each experiments have been repeated three times in triplicate, means and standard deviation of representative experiments are reported. Significance was assessed by one-way ANOVA and Dunnett’s multiple comparisons post-hoc test. *P < 0.05, **P < 0.001, ***P < 0.0001

In a therapeutic scenario, the specificity and selectivity in tumor targeting with minor or insignificant effects on surrounding normal tissues is detrimentally required to maximize anti-tumor effects abating undesirable adverse effects. Therefore, we investigated whether VLIUS treatments might selectively improve delivery in cancer without significant effects in normal cells. Accordingly, analyses were performed with cancer (HT29, HCT116) and normal fibroblast (HF) and endothelial umbilical vein (E926) cells. Of interest, despite the intrinsic peculiarity of each line, VLIUS significantly increases DNA delivery in tumor HT29 and HCT116 lines without any significant effect in normal cells (Fig. 4). The overall results are in support of a different response to VLIUS that occur in tumor cells with respect to normal counterpart providing novel promising insights for the design of more selective anti-tumor treatments.

### VLIUS increases trabectedin efficiency in myxoid sarcoma but not in normal cells

**Fig. 5:**
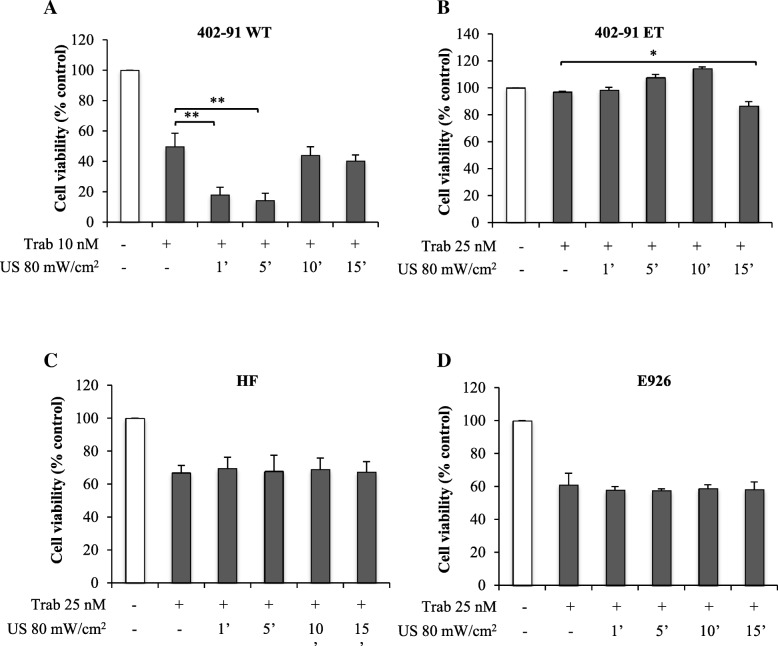
US treatments increases trabectedin uptake in tumor but not in normal cell. **a**, **b**, **c** and **d**, respectively 402–91 WT, 402–91 ET, HF and E926 cells were plated at density of 150,000 cells in 60 mm dish, and the day after treated for 1 h with trabectedin at a concentration of 10 or 25 nM. During the treatment cells were also exposed 1, 5, 10 or 15 min to LIUS source at intensities of 80 mW/cm^2^. Cell vitality was evaluated by Crystal violet assay 48 h after drug removal. Histogram reported percentage of viable cells and error bars represent the standard deviation. Significance was assessed by one-way ANOVA and Dunnett’s multiple comparisons post-hoc test. *P < 0.05, **P < 0.001, ***P < 0.0001

US cavitation increases the permeability to bioactive materials by sonoporation perturbing the cell membrane structures [10, 28, 29]. Accordingly, we asked whether VLIUS might enhance delivery of trabectedin, a marine alkaloid isolated from the tunicate *Ecteinascidia turbinata*, with potent antitumor activity in a wide range of tumors in particular liposarcoma [30–32]. To this aim, myxoid sarcoma 402–91 WT, and trabectedin-resistant 402–91 ET lines and normal HF and E926 lines were treated with trabectedin for 1 h at concentrations of 10 or 25 nM, and thereafter exposed or not to VLIUS for 1, 5, 10 or 15 min with constant frequency (1.0 MHz) and the minimal intensity (80 mW/cm^2^) required to efficiently guarantee the molecule delivery, since lower tested intensities (20 and 40 mW/cm^2^) were ineffective or protective (Additional file 1: Figure S1, A and B). As previously demonstrated by us and other groups [22, 23, 33], trabectedin alone induced 50% of cell death in sensitive 402–91 WT cells, which of interest reached 85% when exposed to VLIUS for 1 and 5 min (Fig. 5a). Noteworthy, the drug treatment followed by VLIUS for 15 min induced a significant 20% of death in 402–91 ET cells (Fig. 5b), suggesting that also in trabectedin-resistant cells VLIUS increase drug uptake inducing cells death (Fig. 5b), albeit not enough to completely revert drug resistance, which might require longer VLUIS time exposure likely due to drug resistance activated pathways in these cells [33]. Of relevance, in normal cell counterparts, trabectedin at higher concentration (25 nM) combined with VLIUS do not induce any significant effect on cell survival in all experimental conditions tested (Fig. 5c, d). These results are in accordance with those shown Fig. 4, where tumor cells might reveal a different behavior to VLIUS than normal cell, providing novel potential features for selective tumor treatments. Overall, our results support that small pores created by VLIUS allow passive diffusion of small molecules, such as trabectidin (761,84 g/mol) into the tumor lesion. This is in agreement with a mechanistically expected effect for low/high US, which the induced pore formation increase the direct cytoplasmic uptake of drugs (whash-in). Contrarily, longer VLIUS treatment (> 5 min) concurs to the exit of small molecules from the cytoplasm (wash-out) as observed with high intensity US [32].

## Discussion

VLIUS has been utilized for cancer therapy studies - sonodynamic therapy, US mediated chemotherapy, US mediated gene delivery and antivascular US therapy [34]. Focused US has been used recently to target DNA-loaded microbubbles located within tumor’s neovasculature to facilitate release of genetic material locally into the tumor [35, 36]. In particular, US has been noted causing the process of sonoporation thus producing transient pores in the cancer cell membranes through which molecules are able to enter the cell [20, 37, 38]. Of interest, successful delivery of genetic material by using microbubbles induces apoptosis in cancer cells and reduces tumor growth [39, 40]. The underlying hypothesis is to deliver genetic materials into specific tumor sites sparing the non-targeted areas [41].

To date there is no widely accepted definition of LIUS and intensity below the 5.0 W/cm^2^ has been recently suggested as maximum value for LUIS application. Of note, the acoustic pressures required to promote gene transfer using microbubbles are usually greater than 0.3 MPa falling into a general classification of moderate US intensities. In these studies, the US-mediated methods for delivery of genetic material was usually accomplished using non-viral and, in a few studies, viral techniques [38, 42]. The non-viral techniques have higher safety with respect to viral vectors but are disadvantaged by the low delivery efficiencies [43]. At the best of our knowledge, the combination of VLIUS and NPs to deliver genetic material or VLIUS and drug to locally deliver chemotherapy into tumors has not been fully explored at power lower than 0.120 W/cm^2^ (i.e. 120 mW/cm^2^). In this regards, this study would highlight the therapeutical potential of our novel device to selectively enhance drug delivery in cancer cells with respect to normal cells.

Of note, our data support that increasing the power of very low intensity non-cavitational US increases significantly the uptake of NPs in both SW872 and SW982 human sarcoma cell lines, considered as representative of less or more aggressive cancer cell lines, respectively. In particular, increasing the intensity up to 120 mW/cm^2^ the uptake significantly increases still maintaining cells vitality without any side effects. Of relevance, the use of an automated tool for the detections of fluorescent dots allowed a fast and precise data elaboration for accurately revealing NPs uptake efficacy. Moreover, VLIUS significantly and selectively increase the delivery of DNA-NPs cargo into tumor cells but not in normal fibroblast and endothelial cells. Of interest, similar tumor specific effects were found when VLIUS were combined to trabectedin in myxoid sarcoma cells, thus opening original scenarios for the development of novel therapeutic treatments.

In addition, relatively few studies focused on biodistribution of the agents and their elimination from the body. Chemotherapeutic agent-loaded microbubbles not destroyed by an US beam which has been localized to a tumor will continue to circulate in the vascular system and may be retained in a major organ (e.g. spleen, followed by decreasing levels respectively in the liver, lung, kidney and other tissues) [44–47]. Recently, it has been reported that the dense and stiff extracellular matrix (ECM) can prevent drug delivery into tumor tissues affecting therapeutic efficacy [48, 49], ECM remodeling and disruption of collagen structure by pulsed-high intensity focused US has been reported as a promising strategy to enhance the deep penetration and tumor targeting in ECM-rich tumor tissues [50]. This issue has still to be explored with VLIUS, insonation of neoplasms with VLIUS is easy to perform, the instruments are relatively inexpensive and the bio-effects in adjacent normal tissues are commonly minimal. Treatment times are prolonged in comparison to those used in high intensity focused US, and treatments can be delivered non-invasively and repeatedly. Multigene approach using a combination of antiangiogenic and pro-apoptotic gene therapies is expected to achieve a synergistic therapeutic response [51].

## Conclusions

Our studies, by adopting three different in vitro experimental tumor models and normal cells and approaching three different therapeutic scenarios, demonstrated VLIUS, as non-invasive and repeatable strategy, to mediate efficient delivery in tumor cells sparing normal tissues. Overall data shed novel lights on the potential application of VLIUS for the design and development of novel therapeutic strategies aiming to efficiently deliver NP loaded cargos or anticancer drugs into more aggressive and unresponsive tumors niche.

## Additional file

Additional file 1: **Figure S1.** US treatments in 402–91 WT cells at lower intensities did not increase trabectedin effect. 402–91 WT cells were plated at density of 150,000 cells in 60 mm dish, and the day after treated for 1 h with trabectedin at a concentration of 10 nM. During the treatment cells were also exposed 1, 5, 10 or 15 min to LIUS source at intensities of 40 or 20 mW/cm2 (A and B). Histogram reported percentage of viable cells and error bars represent the standard deviation. Significance was assessed by one-way ANOVA and Dunnett’s multiple comparisons post-hoc test. *P < 0.05, **P < 0.001, ***P < 0.0001. (JPG 99 kb)

## Abbreviations

DC-Chol: (3β-[N-(N′,N′-dimethylaminoethane)-carbamoyl])-cholesterol
DOPC: Dioleoylphosphocholine
DOPE: Dioleoylphosphatidylethanolamine
DOTAP: 1,2-dioleoyl-3-trimethylammonium-propane
HF: Normal fibroblast
HFt: H-type human ferritin
Ispta: Spatial peak temporal average intensity
LUC: Luciferase reporter
NPs: Nanoparticles
P: Protamine sulfate salt
PEG: Poly(ethylene glycol)
PLB: Passive Lysis Buffer
RGB: Red green blue
R_H_: Hydrodynamic radius
R_W_: Weight ratio
s.d.: Standard deviation
SSD: Source-dish Surface Distance
SUVs: Small unilamellar vesicles
US: Ultrasound
ζ_P_: Zeta-potential
