# Supplementary figures and images for: Very low intensity ultrasounds as a new strategy to improve selective delivery of nanoparticles-complexes in cancer cells
Source: J Exp Clin Cancer Res. 2019 Jan 3;38:1. doi: 10.1186/s13046-018-1018-6 (PMC6318873; doi:10.1186/s13046-018-1018-6)

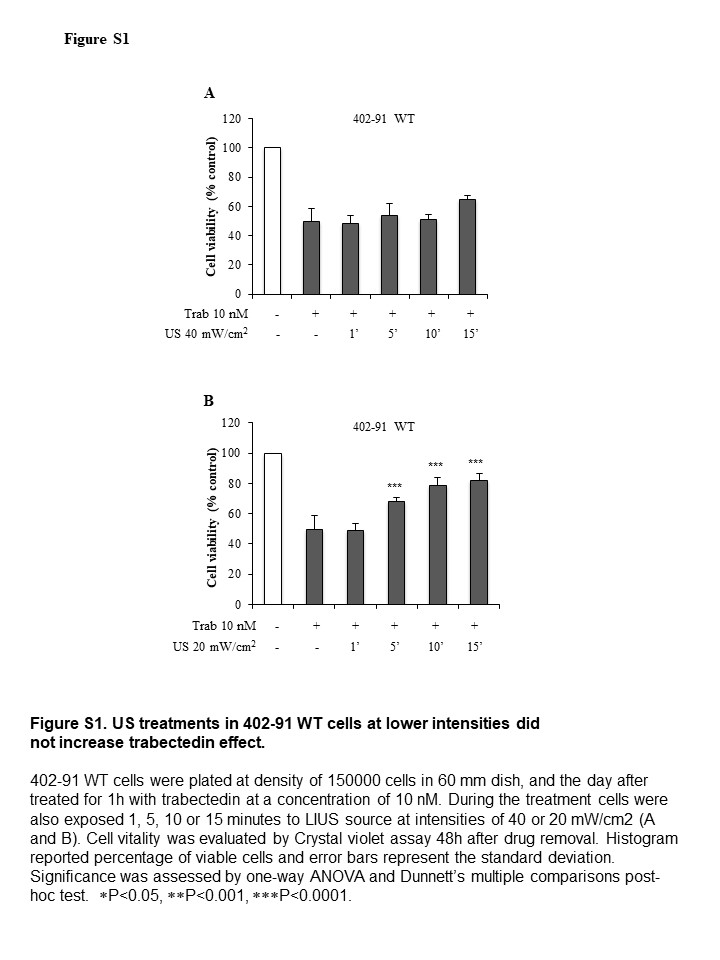

Supplement: Supplementary file 1 — Figure S1. US treatments in 402–91 WT cells at lower intensities did not increase trabectedin effect. 402–91 WT cells were plated at density of 150,000 cells in 60 mm dish, and the day after treated for 1 h with trabectedin at a concentration of 10 nM. During the treatment cells were also exposed 1, 5, 10 or 15 min to LIUS source at intensities of 40 or 20 mW/cm2 (A and B). Histogram reported percentage of viable cells and error bars represent the standard deviation. Significance was assessed by one-way ANOVA and Dunnett’s multiple comparisons post-hoc test. *P < 0.05, **P < 0.001, ***P < 0.0001. (JPG 99 kb) [file 13046_2018_1018_MOESM1_ESM.jpg]
